# Oral relugolix for the treatment of advanced prostate cancer

**DOI:** 10.1093/oncolo/oyag004

**Published:** 2026-01-28

**Authors:** Ronald Chow, Amanda Hird, Michael Lock

**Affiliations:** Temerty Faculty of Medicine, University of Toronto, Toronto, ON M5S 3K3, Canada; Verspeeten Family Cancer Centre, University of Western Ontario, London, ON N6A 5W9, Canada; Centre for Evidence-Based Medicine, University of Oxford, Oxford, OX2 6HT, United Kingdom; Temerty Faculty of Medicine, University of Toronto, Toronto, ON M5S 3K3, Canada; Verspeeten Family Cancer Centre, University of Western Ontario, London, ON N6A 5W9, Canada

## Introduction

Relugolix is the first oral gonadotropin-releasing hormone (GnRH) antagonist developed specifically to address issues with traditional androgen deprivation therapy (ADT). With the publication of the HERO trial, where investigators compared oral relugolix and leuprolide in men with advanced prostate cancer.^1^ This multinational randomized trial is hailed as “pivotal” and “transformative.”^2^^,^^3^ The trial is hailed for demonstrating superior efficacy and safety with a 96.7% sustained testosterone suppression compared to 88.8% with leuprolide; plus a faster testosterone suppression and recovery. One of the principal reasons HERO has been regarded as a landmark study is the reported 54% reduction in major adverse cardiovascular events with relugolix, a benefit that has not been confirmed with other androgen-deprivation agents in dedicated cardiovascular outcome trials.^4^ MACE was defined as non-fatal myocardial infarction, non-fatal stroke or cardiovascular mortality. The number needed to treat (NNT) to avoid one MACE event at 48 weeks was an impressive 31. This is particularly important as cardiovascular disease is a leading cause of death in men with prostate cancer with ADT being an important factor in patients with pre-existing cardiovascular disease. In this subgroup, the risk was 4.9 times lower if relugolix was used (3.6% vs 17.8%). However, one has to wonder—how stable are these findings? Could a few event changes erase the statistical significance?

When we read a trial with a *P* < .05 and nod to “statistically significant,” we sometimes forget just how brittle those results might be. A handful of patient outcomes shifted one way or another and the results can tip over. That’s where the fragility index (FI) comes in: it tells us how many event flips would undo a trial’s claim of significance. In cancer trials, where differences are often marginal and events uncommon, the FI offers useful intuition beyond *P* values and confidence intervals. Median FI has been reported to be only 2; if 2 patients were to have had a different outcome, the result would go from significant to nonsignificant results.^5^

In this article, we analyze the published results to compute the FI for the two pivotal outcomes—sustained castration and major cardiovascular events. Our objective is to assess the robustness of the results through a re-analysis of the previously-published aggregate data, so readers can see not just the results but also the durability of the results.

## Methods

We extracted data directly from the published trial, which compared relugolix (*n* = 622) and leuprolide (*n* = 308). We extracted the counts of patients who experienced:

1. Sustained castration through 48 weeks
2. Major adverse cardiovascular events (MACE) during treatment

For each endpoint, we constructed a 2 × 2 contingency table using the reported event and non-event counts. The FI was calculated using the Walsh et al. approach.^6^ Briefly, FI is defined as the smallest number of individual outcome status changes (from event to non-event) needed to render a two-sided Fisher’s exact test nonsignificant (*P* > .05).

Analysis was conducted using StataBE 18.0. All data are publicly available in aggregate form, and therefore no ethics approval required.

## Results

Table 1 presents the 2 × 2 table for the two main dichotomous outcomes. For the outcome of sustained castration, 601/622 patients on relugolix achieved the outcome, compared to 274/308 in the leuprolide group. FI was 15. For the outcome of major adverse cardiovascular events, 18/622 patients on relugolix achieved the outcome, compared to 19/308 in the leuprolide group. FI was 3.

**Table 1.** Fragility index table.

Outcome	Group	Events	Non-events	Fragility index
Sustained castration	Relugolix	601 (97%)	21 (3%)	15
	Leuprolide	274 (89%)	34 (11%)	
Major adverse cardiovascular events	Relugolix	18 (2%)	604 (98%)	3
	Leuprolide	19 (6%)	289 (94%)	

## Discussion

This is the first study to report robustness of the HERO trial data. The outcome of sustained castration has very robust data, but the outcome major adverse cardiovascular events is much more fragile. Therefore, HERO study alone may be insufficient to change clinical practice and decisions must be made in context. Supporting factors include biological plausibility, consistent direction of effect across subgroups (especially in patients with prior cardiovascular disease), quality of the trial methodology, FI level is in keeping with other practice changing oncological studies, and magnitude of effect (a 54% reduction in MACE is a clinically meaningful effect and is a clinically important endpoint. Of note, MACE events were adjudicated by an independent committee that confirmed cardiovascular events blinded to the randomization arm and it was a secondary endpoint of the trial, and therefore not necessarily powered to detect significant differences). Cautionary factors include statistical vulnerability (a fragility index of 3 is tenuous especially when the HERO investigators report a 10% termination rate in both arms) and the need for replication. In terms of confirmatory trials, there are three phase IV trials: First, RElugolix VErsus LeUprolide Cardiac Trial (REVELUTION) (NCT 05320406) is the most direct confirmatory study and aims to assess cardiovascular toxicity and identify biomarkers for individualized risk assessment in this patient group.^7^ Second, the OPTYX Study (NCT05467176) is a prospective observational study of 999 men assessing long-term safety and effectiveness.^8^ Third, the FAERS Database is reporting no new major cardiovascular safety signals which reinforces the HERO safety profile, though not directly addressing MACE.^9^

As previously mentioned, oncology literature tends to have a median FI of 2. The primary efficacy endpoint of sustained castration is therefore extremely robust. In comparison, the outcome major adverse cardiovascular events is less robust (a few patients coded differently could overturn the conclusion), but still in keeping with what is expected in oncologic literature. It is important to mention, however, that the prevalence is rare, and therefore with low counts, a low FI is nearly unavoidable.

Finally, it is important to mention that FI does not take into account biological plausibility or consistency across subgroups. It should therefore be viewed as a supplementary metric rather than a substitute for traditional safety data interpretation.

In conclusion, the results of the relugolix are significant and very robust in terms of sustained castration. In terms of selecting this agent specifically for cardiovascular reasons, this analysis supports its practice-changing impact, especially for patients with pre-existing events, given the totality of the supporting context such as the biological rationale, consistent direction of effect, magnitude of impact, quality of the trial methodology, FI level is in keeping with other practice changing oncological studies. However, clinicians should remain cautious as the results are statistically fragile and confirmatory studies are required.
